# Association of Physician Organization–Affiliated Political Action Committee Contributions With US House of Representatives and Senate Candidates’ Stances on Firearm Regulation

**DOI:** 10.1001/jamanetworkopen.2018.7831

**Published:** 2019-02-22

**Authors:** Jeremiah D. Schuur, Hannah Decker, Olesya Baker

**Affiliations:** 1 Department of Emergency Medicine, Rhode Island Hospital, Providence; 2 Department of Emergency Medicine, Alpert Medical School of Brown University, Providence, Rhode Island; 3 Emory School of Medicine, Atlanta, Georgia; 4 Department of Emergency Medicine, Brigham and Women’s Hospital, Boston, Massachusetts

## Abstract

**Importance:**

Many physician professional organizations have endorsed public policies, such as expanded background checks, to reduce firearm-related injury. It is not known whether physician organizations’ political giving aligns with these policy endorsements.

**Objectives:**

To compare physician organization–affiliated political action committee (PAC) campaign contributions with US House of Representatives and Senate candidates’ stances on firearm safety policies and analyze whether physician organization endorsement of firearm safety policies is associated with contribution patterns.

**Design, Setting, and Participants:**

This cross-sectional study compared contributions from the 25 largest physician organization–affiliated PACs during the 2016 election cycle (January 1, 2014, to December 31, 2016) with US House of Representatives and Senate candidate support for firearm regulation. Physician organization endorsement of firearm safety policies was defined by endorsement of the 2015 Firearm-Related Injury and Death in the United States: A Call to Action From 8 Health Professional Organizations and the American Bar Association.

**Main Outcomes and Measures:**

Contributions to US House of Representatives and Senate candidates by stance on firearm safety legislation measured by (1) voting history on US Senate Amendment (SA) 4750, which proposed background check expansion; (2) cosponsorship of US House Resolution (HR) 1217, which sought to expand background checks and strengthen the national criminal background check system; and (3) an A rating (vs not A) by the National Rifle Association Political Victory Fund (NRA-PVF), a measure of overall candidate support for firearm regulation.

**Results:**

This study examined the 25 largest physician organization–affiliated PACs during the 2016 election cycle. Twenty of 25 PACs (80%) contributed more in total to incumbent Senate candidates who voted against SA 4750 (n = 21) than to those who voted for it (n = 8), and 24 of 25 PACs (96%) contributed more in total to incumbent US House of Representatives candidates who did not cosponsor HR 1217 (n = 227) than to those who cosponsored it (n = 166). A total of 21 of 25 PACs (84%) contributed more total dollars to US House of Representatives and Senate candidates rated A by the NRA-PVF (n = 386) than to those not rated A (n = 546). Twenty-four of 25 PACs (96%) contributed to a greater proportion of candidates rated A by the NRA-PVF than candidates not rated A. Among PACs whose affiliated organizations endorsed the Call to Action, 8 of 9 (89%) supported a greater proportion of candidates rated A by the NRA-PVF than candidates not rated A, whereas 16 of 16 PACs affiliated with nonendorsing organizations supported a greater proportion of candidates rated A by the NRA-PVF. After adjustment for other political factors, the 9 PACs that endorsed the Call to Action had a lower likelihood of donating to NRA-PVF A-rated candidates compared with PACs that did not endorse the Call to Action (odds ratio, 0.76; 95% CI, 0.58-0.99; *P* = .04).

**Conclusions and Relevance:**

Physician organization–affiliated PACs included in this study donated more funds to more US House of Representatives and Senate candidates who oppose firearm safety policies than to candidates in support of such policies. Although endorsement of the Call to Action was associated with a lower likelihood of donating to candidates who oppose firearm safety policies, the overall pattern was not consistent with professional societies’ advocacy for firearm safety.

## Key Points

**Question:**

Do US physician organization–affiliated political action committees make campaign contributions to US House of Representatives and Senate candidates whose stances on firearm regulation align with their own?

**Findings:**

This cross-sectional study of the 25 largest physician organization–affiliated political action committees during the 2016 election cycle compared candidate contributions from the committees and US House of Representatives and Senate candidates’ support for firearm regulation. Physician organization–affiliated political action committees contributed more money and to a higher proportion of candidates who voted against expanding firearm background checks, an evidence-based policy to reduce firearm-related injury and death.

**Meaning:**

The findings suggest that contribution patterns of most physician organization–affiliated political action committees are inconsistent with their policy recommendations for evidence-based firearm regulation and may pose a barrier for effective advocacy on this issue.

## Teaser

This cross-sectional study compares physician organization–affiliated political action committee (PAC) campaign contributions with US House of Representatives and Senate candidate stance on firearm safety policies to examine whether physician organization endorsement of firearm safety policies is associated with contribution patterns.

## Introduction

Firearm violence, the third leading cause of injurious death in the United States, is an increasing public health problem. Firearms were responsible for 39 773 deaths in 2017.^1^ Firearm-related injuries lead to significant health care utilization, amounting to $25 billion in emergency department and inpatient hospitalization charges from 2006 to 2014.^2,3^

In response to the magnitude of firearm violence and numerous high-profile mass shootings, many health care organizations have endorsed policy recommendations to reduce firearm-related morbidity and mortality. In 2015, a total of 8 health care professional organizations and the American Bar Association wrote Firearm-Related Injury and Death in the United States: A Call to Action From 8 Health Professional Organizations and the American Bar Association (hereafter referred to as the Call to Action), which was later endorsed by more than 40 additional organizations.^4^ The Call to Action includes recommendations for universal background checks for firearm purchases, removing laws that prohibit physicians from discussing firearm safety with patients, promotion of firearm-related research, restrictions on military-style assault weapons, and more recommendations.

One way that physician professional organizations can exert influence on policy at national and state levels is through their affiliated political action committees (PACs). The role of a PAC is to raise and spend money to elect candidates whose policy stances align with their own or to oppose candidates with differing stances. Combined, health care professional–affiliated PACs contributed a mean of $25.4 million dollars per 2-year legislative cycle to federal campaigns during 2012 to 2016, accounting for more than 5% of all PAC contributions in this period.^5^ Although analysis in 1994 found that the American Medical Association’s (AMA’s) affiliated PAC contributed more money to candidates who opposed handgun control than to those in support,^6^ to our knowledge, no contemporary research has described the association between physician organization–affiliated PAC contributions and candidates’ stances on firearm regulation and whether these stances align with the policies supported by the professional organizations.

To evaluate this question, we analyzed contributions from the 25 largest physician organization–affiliated PACs to US House of Representatives and Senate candidates (hereafter referred to as candidates) during the 2016 election cycle and compared these with candidate support for firearm regulation. We assessed candidates’ support for firearm regulation by analyzing voting records on key firearm safety bills and ratings by the National Rifle Association Political Victory Fund (NRA-PVF), the PAC affiliated with the NRA. We also analyzed whether physician organization endorsement of the 2015 Call to Action was associated with contribution patterns and hypothesized that endorsing organizations would be less likely to contribute to candidates rated A by the NRA-PVF.

## Methods

We conducted a retrospective, cross-sectional study of the association between contributions from the 25 largest physician organization–affiliated PACs to candidates in the 2016 federal congressional general election (January 1, 2014, to December 31, 2016) and candidate support for firearm regulation. The largest 25 PACs were defined by total campaign contributions in the 2016 federal congressional general election cycle. We assessed candidates’ stances on firearm regulation using the following measures: voting records on key firearm safety legislation proposed during the 114th congressional term and ratings given by the NRA-PVF. The study was deemed exempt from institutional review board review by Brigham and Women’s Hospital. This report follows Strengthening the Reporting of Observational Studies in Epidemiology (STROBE) reporting guideline for cross-sectional studies.

### Data Sources

We obtained data on PAC candidate contributions from Open Secrets,^5,7^ an independent, nonpartisan website that aggregates data from the Federal Election Commission. We obtained data on legislator voting records from the US Congress website.^8^ We obtained other candidate data, including party affiliation, state, district, incumbent status, NRA-PVF ratings, general election vote share (percentage of vote received), and election outcome, from Project VoteSmart),^9^ a nonpartisan, independent organization that aggregates information on political candidates. We determined physician organization public policies on firearms policy by searching their websites (eAppendix 1 in the Supplement).

### Exposure

The exposures we used to determine candidates’ support or opposition to firearm safety policies included support for specific legislation in the US Senate and US House of Representatives in the 114th US Congress that proposed expanding background checks and candidate ranking by the NRA-PVF. We chose background checks because research demonstrates that they are associated with reducing firearm violence,^10^ and most of the physician specialty societies that have public positions on firearm regulations endorse them (eAppendix 1 in the Supplement). US Senate Amendment 4750 (SA 4750) sought to expand background checks for firearm transactions and was the most comprehensive background check expansion bill that received a vote during the study period, getting voted down in 2016.^11^ Because no House bill featuring background checks underwent a vote in the 114th US Congress, we evaluated US House Resolution 1217 (HR 1217), the bill supporting background checks that received the most cosponsorship. Cosponsorship is a formal endorsement of a congressional bill and is recorded in the *Congressional Record*. Introduced in 2015, HR 1217 aimed to extend background checks to all commercial firearm sales, strengthen state cooperation with the national criminal background check system, and authorize a commission to study mass violence.^12^

An alternate measure of candidate support for expanding firearm safety policies was candidate rating by the NRA-PVF. The NRA-PVF is a single-issue PAC that ranks political candidates, irrespective of party affiliation, on their support for the NRA’s mission. The NRA-PVF has opposed expansion of background checks, limitations on assault weapons, and both SA 4750 and HR 1217.^13,14,15,16,17,18^ Ratings range from A+ to F (eAppendix 2 in the Supplement). We grouped candidates rated A (A+, A, A−, or Aq [an A rating given because of a questionnaire vs a voting record]) vs not A (B+ through F) by the NRA-PVF because the ratings are bimodal (94% are A or F), and A ratings are only given to politicians who have consistently voted with the NRA-PVF’s position.

To account for the association of other political factors with contributions from PACs to candidates, we also assessed incumbency, vote share, and party affiliation. Historically, PACs are more likely to support candidates who are more likely to win because it improves their influence, and incumbency is the strongest determinant of electoral success.^7,19^ Vote share tracks closely with preelection polling that is available to PACs. Party affiliation is linked to candidates’ positions on other health care–related topics that may influence PAC contributions, including physician reimbursement, malpractice reform, and repeal of the Patient Protection and Affordable Care Act.

### Outcome Measures

The primary outcome was total contributions to candidates by support or opposition to firearm safety legislation, as measured by support of SA 4750 or HR 1217. The secondary outcome was proportion of and total contributions to candidates rated A (vs not A) by the NRA-PVF, a measure of overall candidate support for expanding firearm regulation.

### Statistical Analysis

For the analysis of support of firearm safety bills, we included 2016 general election incumbent candidates who had legislative records on the bills. In the US Senate, there were 29 incumbent candidates with an SA 4750 voting record; in the US House of Representatives, there were 394 incumbent candidates with an opportunity to cosponsor HR 1217. We computed total and mean contributions stratified by candidate support of legislation for each PAC. Mean contribution was calculated by including only the candidates who received contributions from that PAC because most candidates received no contribution, which would skew the mean toward zero.

For the analysis of NRA-PVF ratings and contributions, we included all 2016 general election candidates who were rated by the NRA-PVF (932 of 1345). We excluded unrated candidates because their position on firearm policy was not clear to the NRA-PVF. These candidates were largely lower-profile candidates who were unlikely to win and received low vote shares (median vote share for nonrated candidates was 29.2%; highest was 38.4%). As such, they rarely received physician PAC contributions. We computed total and mean contributions stratified by candidate NRA-PVF rating (A vs not A) for each PAC, similarly including only those candidates who received any contribution in each PAC’s mean. We also computed the proportion of candidates receiving PAC support stratified by NRA-PVF ratings. This proportion was computed using only candidates who won the election or received the second highest vote share (N=818) to avoid including candidates who received no contribution because of low likelihood of winning. We did not calculate *P* values or report 95% CIs for these descriptive results because this was an enumeration of the data.

We constructed a multiple variable regression analysis to examine the association between society endorsement of the Call to Action and associated PAC contribution to candidates by NRA-PVF rating, accounting for other political factors that could influence PAC contributions. Our analytic sample was again candidates who won or received the second highest vote share. We constructed a logistic regression with the dependent variable of a PAC contribution (yes or no) and independent indicator variables for A vs not A rating by NRA-PVF, PAC endorsement of the Call to Action, and interaction of NRA-PVF rating and endorsement of Call to Action dummies. We calculated adjusted odds ratios accounting for vote share, incumbency, party affiliation, and legislative branch. The SEs were clustered at PAC and candidate levels (eAppendix 3 in the Supplement). All analyses were performed with Stata statistical software, version 13.1 (StataCorp). A 2-sided *P* < .05 indicates statistical significance.

## Results

This study examined the 25 largest physician organization–affiliated PACs during the 2016 election cycle. All health care professional–related PACs contributed a total of $23.7 million dollars during the 2016 election cycle. The 25 largest physician-affiliated PACs contributed 57% of this sum ($13.6 million).

### Association With Congressional Bills

The US Senate voted 44 for and 56 against SA 4750, an amendment that would have expanded background checks. Table 1 details physician organization–affiliated PAC support of the 29 Senate incumbents running for reelection and the association with their vote on SA 4750. Overall, the 25 PACs contributed $500 000 more to Senate candidates who voted against SA 4750 than to those who voted for it ($1 025 500 vs $525 500). A total of 20 of 25 PACs (80%) contributed more in total to Senate candidates who voted against SA 4750 than to those who voted for it.

**Table 1.** Contributions From Physician Organization–Affiliated PACs to US Senate Candidates Compared With Voting Record on SA 4750^a^

Physician Organization–Affiliated PAC	Total Contributions				Mean Contribution		
	Candidates Voting for SA 4750, $	Candidates Voting Against SA 4750, $	Difference, $	Contributions to Candidates Voting for SA 4750, %	Candidates Voting for SA 4750, $ (No. of Candidates)	Candidates Voting Against SA 4750, $ (No. of Candidates)	Difference, $
Total	525 500	1 025 500	−500 000	34	4821 (8)	4498 (21)	323
American Association of Orthopaedic Surgeons	27 000	111 000	−84 000	20	4500 (6)	5842 (19)	−1342
American Medical Association^b^	58 000	109 000	−51 000	35	7250 (8)	7786 (14)	−535
American Society of Anesthesiologists	24 500	37 500	−13 000	40	4687 (8)	4083 (6)	604
American College of Emergency Physicians^b^	44 500	82 998	−38 498	35	7416 (6)	5533 (15)	1883
American College of Radiology	24 500	72 000	−47 500	25	4900 (5)	5538 (13)	−638
American Academy of Dermatology	34 000	73 000	−39 000	32	5666 (6)	5615 (13)	51
American Academy of Ophthalmology	15 500	34 500	−19 000	31	3875 (4)	4312 (8)	−437
American College of Surgeons^b^	36 500	62 500	−26 000	37	6083 (6)	5208 (12)	875
American Academy of Family Physicians^b^	34 000	40 500	−6500	46	5666 (6)	4500 (9)	1166
American Congress of Obstetricians & Gynecologists^b^	29 000	23 000	6000	56	4833 (6)	2300 (10)	2533
American Academy of Neurology^b^	13 500	20 500	−7000	40	2250 (6)	2928 (7)	−678
American College of Cardiology	28 500	40 000	−11 500	42	5700 (5)	4444 (9)	1255
American Association of Oral and Maxillofacial Surgery	22 000	32 000	−10 000	41	3200 (10)	5500 (4)	−2300
American Psychiatric Association^b^	19 500	17 500	2000	53	3900 (5)	2187 (8)	1713
College of American Pathologists	20 500	34 000	−13 500	38	5125 (4)	3091 (11)	2034
American Society of Plastic Surgeons	13 500	87 000	−73 500	13	4500 (3)	5800 (15)	−1300
American College of Rheumatology	17 500	29 500	−12 000	37	3500 (5)	3278 (9)	222
American Academy of Otolaryngology	13 000	6000	7000	68	3250 (4)	3000 (2)	250
Society of Thoracic Surgeons^b^	14 500	12 500	2000	54	2900 (6)	2083 (5)	816
American Society for Radiation Oncology	18 500	14 500	4000	56	4625 (4)	4833 (3)	−208
American College of Physicians Services^b^	8500	11 500	−3000	42	2833 (3)	2300 (5)	533
American Association of Neurologic Surgeons	8500	29 500	−21 000	22	4250 (2)	3278 (9)	972
American Society of Interventional Pain Physicians	0	17 500	−17 500	0	0 (0)	5833 (3)	−5833
National Association of Spine Specialists	0	23 500	−23 500	0	0 (0)	3916 (6)	−3916
Society for Vascular Surgery	0	4000	−4000	0	0 (0)	1000 (4)	−1000

Abbreviations: PAC, political action committee; SA, US Senate Amendment.

^a^ From 2014 to 2016, including US Senate incumbents seeking reelection in 2016.

^b^ Endorsed the 2015 Firearm-Related Injury and Death in the United States: A Call to Action From 8 Health Professional Organizations and the American Bar Association.^4^

In the US House of Representatives, 189 representatives cosponsored HR 1217, a bill to expand background checks, and 246 did not. Table 2 details PAC support of 394 incumbent House candidates and the association with cosponsorship of HR 1217. Overall, the 25 PACs contributed $2 878 675 more to candidates who did not cosponsor HR 1217 than to cosponsors ($6 130 775 vs $3 252 100). A total of 24 of 25 PACs (96%) contributed more in total to House candidates who did not cosponsor HR 1217 than those who cosponsored it.

**Table 2.** Contributions From Physician Organization–Affiliated PACs to US House of Representative Candidates Compared With Cosponsorship of HR 1217^a^

Physician Organization-Affiliated PAC	Total Contributions				Mean Contribution		
	Cosponsor of HR 1217, $	Not Cosponsor of HR 1217, $	Difference, $	Contributions to Candidates Cosponsoring HR 1217, %	Cosponsor of HR 1217, $ (No. of Candidates)	Not Cosponsor of HR 1217, $ (No. of Candidates)	Difference, $
Total	3 252 100	6 130 775	−2 878 675	35	4234 (133)	4379 (196)	−144
American Association of Orthopaedic Surgeons	374 550	699 525	−324 975	35	5852 (64)	5465 (128)	387
American Medical Association^b^	348 500	664 000	−315 500	34	3707 (94)	4311 (154)	−604
American Society of Anesthesiologists	315 400	663 700	−348 300	32	5256 (60)	6033 (110)	−776
American College of Emergency Physicians^b^	227 955	625 385	−397 430	27	3352 (68)	5255 (119)	−1902
American College of Radiology	173 500	440 500	−267 000	28	4565 (38)	5438 (81)	−872
American Academy of Dermatology	241 500	353 000	−111 500	41	5488 (44)	4835 (73)	653
American Academy of Ophthalmology	116 500	312 500	−196 000	27	4660 (25)	4340 (72)	319
American College of Surgeons^b^	158 000	292 000	−134 000	35	5642 (28)	5214 (56)	428
American Academy of Family Physicians^b^	209 200	206 500	2700	50	5102 (41)	4393 (47)	708
American Congress of Obstetricians & Gynecologists^b^	134 000	144 500	−10 500	48	3268 (41)	3010 (48)	257
American Academy of Neurology^b^	147 000	203 000	−56 000	42	4083 (36)	3561 (57)	521
American College of Cardiology	119 000	193 700	−74 700	38	6611 (18)	4724 (41)	1886
American Association of Oral and Maxillofacial Surgery	101 500	198 000	−96 500	34	3274 (31)	3882 (51)	−608
American Psychiatric Association^b^	116 000	138 500	−22 500	46	4142 (28)	3462 (40)	680
College of American Pathologists	80 950	164 250	−83 300	33	3435 (24)	3352 (49)	83
American Society of Plastic Surgeons	31 500	80 200	−48 700	28	2100 (15)	2864 (28)	−764
American College of Rheumatology	57 500	89 000	−31 500	39	4107 (14)	3560 (25)	547
American Academy of Otolaryngology	56 500	98 000	−41 500	37	4036 (14)	3161 (31)	874
Society of Thoracic Surgeons^b^	59 000	71 500	−12 500	45	2565 (23)	1932 (37)	632
American Society for Radiation Oncology	47 000	74 500	−27 500	39	3133 (15)	2865 (26)	267
American College of Physicians Services^b^	68 000	70 000	−2000	49	3578 (19)	2916 (24)	662
American Association of Neurologic Surgeons	15 500	100 500	−85 000	13	2716 (37)	3600 (5)	−883
American Society of Interventional Pain Physicians	0	127 500	−127 500	0	0 (0)	6071 (21)	−6071
National Association of Spine Specialists	23 500	57 500	−34 000	29	2350 (10)	2300 (25)	50
Society for Vascular Surgery	30 000	63 000	−33 000	32	2142 (14)	3150 (20)	−1007

Abbreviations: HR, US House Resolution; PAC, political action committee.

^a^ From 2014 to 2016, including US House of Representatives incumbents seeking reelection in 2016.

^b^ Endorsed 2015 Firearm-Related Injury and Death in the United States: A Call to Action From 8 Health Professional Organizations and the American Bar Association.^4^

### Association With NRA-PVF Ratings and Contributions

The NRA-PVF gave 379 House and Senate candidates A ratings, 13 B ratings, 8 C ratings, 35 D ratings, and 474 F ratings. Table 3 details PAC financial support of congressional candidates and the association with NRA-PVF rating. Overall, the 25 largest physician organization–affiliated PACs gave $5.6 million to candidates rated A by the NRA-PVF and $4.1 million to candidates not rated A, a difference of $1.5 million. Twenty-one of 25 PACs (84%) contributed more in total to candidates who were rated A by the NRA-PVF than to candidates who were not A rated. Eight of the 25 PACs (32%) contributed more per candidate to candidates rated A by the NRA-PVF than to candidates who were not rated A.

**Table 3.** Physician Organization–Affiliated PACs’ US House of Representatives and Senate Campaign Contributions Compared With Candidate NRA-PVF Rating^a^

Physician Organization–Affiliated PAC	Total Contributions				Mean Contributions		
	Candidates Rated B-F by the NRA-PVF, $	Candidates Rated A by the NRA-PVF, $	Difference, $	Contributions to Candidates Rated B-F by the NRA-PVF, %	Candidates Rated B-F by the NRA-PVF, $ (No. of Candidates)	Candidates Rated A by the NRA-PVF, $ (No. of Candidates)	Difference, $
Total	4 089 550	5 561 375	−1 471 825	42	4262 (176)	4459 (209)	−198
American Association of Orthopaedic Surgeons	416 500	597 125	−180 625	41	5480 (76)	5331 (112)	148
American Medical Association^b^	420 500	574 500	−154 000	42	4163 (101)	4488 (128)	−324
American Society of Anesthesiologists	380 600	594 700	−214 100	39	5437 (70)	6194 (96)	−757
American College of Emergency Physicians^b^	344 500	716 900	−372 400	32	4053 (85)	5233 (137)	−1178
American College of Radiology	190 000	382 000	−192 000	33	4634 (41)	5617 (68)	−983
American Academy of Dermatology	284 000	306 500	−22 500	48	5259 (54)	4715 (65)	543
American Academy of Ophthalmology	128 500	239 000	−110 500	35	4431 (29)	4267 (56)	163
American College of Surgeons^b^	185 500	257 500	−72 000	42	5621 (33)	5597 (46)	23
American Academy of Family Physicians^b^	357 700	262 000	95 700	58	5338 (67)	4678 (56)	660
American Congress of Obstetricians & Gynecologists^b^	247 000	179 500	67 500	58	3383 (73)	3205 (56)	178
American Academy of Neurology^b^	142 500	135 000	7500	51	3653 (39)	3139 (43)	514
American College of Cardiology	133 500	142 200	−8700	48	5804 (23)	4587 (31)	1217
American Association of Oral and Maxillofacial Surgery	109 000	180 000	−71 000	38	3406 (32)	4285 (42)	−879
American Psychiatric Association^b^	154 500	172 500	−18 000	47	3678 (42)	3317 (52)	361
College of American Pathologists	103 750	118 250	−14 500	47	3289 (32)	3111 (38)	177
American Society of Plastic Surgeons	45 500	119 700	−74 200	28	2527 (18)	3627 (33)	−1099
American College of Rheumatology	66 000	71 500	−5500	48	3763 (19)	3300 (20)	463
American Academy of Otolaryngology	65 500	73 000	−7500	47	3852 (17)	3173 (23)	679
Society of Thoracic Surgeons^b^	69 500	54 500	15 000	56	2482 (28)	2595 (21)	−113
American Society for Radiation Oncology	55 000	57 000	−2000	49	2894 (19)	2850 (20)	44
American College of Physicians Services^b^	154 500	172 500	−18 000	47	3678 (42)	3317 (52)	361
American Association of Neurologic Surgeons	28 500	77 500	−49 000	27	3166 (9)	2672 (29)	494
American Society of Interventional Pain Physicians	10 000	95 000	−85 000	10	10 000 (1)	5937 (16)	4062
National Association of Spine Specialists	26 500	37 500	−11 000	41	2409 (11)	2343 (16)	65
Society for Vascular Surgery	32 000	40 000	−8000	44	2133 (15)	3076 (13)	−943

Abbreviations: NRA-PVF, National Rifle Association Political Victory Fund; PAC, political action committee.

^a^ From 2014 to 2016, including US House of Representatives and US Senate candidates with NRA-PVF ratings.

^b^ Endorsed 2015 Firearm-Related Injury and Death in the United States: A Call to Action From 8 Health Professional Organizations and the American Bar Association.^4^

Table 4 details the proportion of House and Senate candidates receiving PAC support stratified by NRA-PVF ratings. A total of 24 of 25 PACs (96%) contributed to a greater proportion of the candidates who were rated A by the NRA-PVF than candidates who were not A rated.

**Table 4.** Physician Organization–Affiliated PACs’ Relative Support of Candidates Rated A vs Not Rated A by NRA-PVF

Physician Organization–Affiliated PAC	Support of Candidates by NRA-PVF Rating		
	Candidates Rated A by the NRA-PVF, No./Total No. (%)	Candidates Rated B-F by the NRA-PVF, No./Total No. (%)	Difference, %
American Association of Orthopaedic Surgeons	109/348 (31.3)	76/470 (16.2)	15.2
American Medical Association^a^	126/348 (36.2)	101/470 (21.5)	14.7
American Society of Anesthesiologists	92/348 (26.4)	70/470 (14.9)	11.5
American College of Emergency Physicians^a^	135/348 (38.8)	85/470 (18.1)	20.7
American College of Radiology	67/348 (19.3)	41/470 (8.7)	10.5
American Academy of Dermatology	63/348 (18.1)	54/470 (11.5)	6.6
American Academy of Ophthalmology	55/348 (15.8)	29/470 (6.2)	9.6
American College of Surgeons^a^	44/348 (12.6)	33/470 (7.0)	5.6
American Academy of Family Physicians^a^	54/348 (15.5)	67/470 (14.3)	1.2
American Congress of Obstetricians & Gynecologists^a^	55/348 (15.8)	73/470 (15.5)	0.3
American Academy of Neurology^a^	42/348 (12.1)	39/470 (8.3)	3.8
American College of Cardiology	30/348 (8.6)	23/470 (4.9)	3.7
American Association of Oral and Maxillofacial Surgery	42/348 (12.1)	32/470 (6.8)	5.2
American Psychiatric Association^a^	50/348 (14.4)	42/470 (9.0)	5.4
College of American Pathologists	38/348 (10.9)	32/470 (6.8)	4.1
American Society of Plastic Surgeons	32/348 (9.2)	18/468 (3.9)	5.3
American College of Rheumatology	18/348 (5.2)	19/468 (4.1)	1.1
American Academy of Otolaryngology	21/348 (6.0)	17/470 (3.6)	2.4
Society of Thoracic Surgeons^a^	20/348 (5.8)	28/470 (6.0)	-0.2
American Society for Radiation Oncology	19/348 (5.5)	19/468 (4.1)	1.4
American College of Physicians Services^a^	30/348 (8.6)	26/470 (5.5)	3.1
American Association of Neurologic Surgeons	28/348 (8.1)	9/470 (1.9)	6.1
American Society of Interventional Pain Physicians	16/348 (4.6)	1/468 (0.2)	4.4
National Association of Spine Specialists	16/348 (4.6)	11/470 (2.4)	2.3
Society for Vascular Surgery	12/348 (3.5)	15/470 (3.2)	0.2

Abbreviations: NRA-PVF, National Rifle Association Political Victory Fund; PAC, political action committee.

^a^ Endorsed 2015 Firearm-Related Injury and Death in the United States: A Call to Action From 8 Health Professional Organizations and the American Bar Association.^4^

### Association With the 2015 Call to Action

The Figure shows PAC contribution patterns comparing total donations to NRA-PVF A-rated candidates with those to non–A-rated candidates and whether each organization endorsed the 2015 Call to Action. Among the 9 PACs whose affiliated organizations endorsed the Call to Action, 8 (89%) supported a greater proportion of candidates rated A by the NRA-PVF than candidates not rated A, whereas all 16 PACs affiliated with nonendorsing organizations supported a greater proportion of candidates rated A by the NRA-PVF (Table 4). After adjustment for other political factors, the 9 PACs that endorsed the Call to Action had a lower likelihood of donating to NRA-PVF A-rated candidates compared with PACs that did not endorse the Call to Action (odds ratio, 0.76; 95% CI, 0.58-0.99; *P* = .04) (eAppendix 3 in the Supplement).

**Figure.**
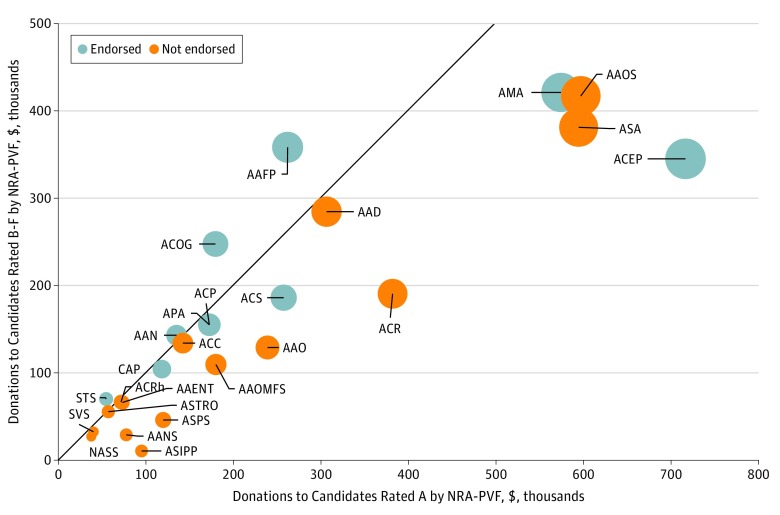
Physician Organization Political Action Committee Contributions Compared With National Rifle Association Political Victory Fund (NRA-PVF) Rating, 2016 Line represents equality of donations to A-rated and non–A-rated candidates. Circle sizes are proportional to total political action committee contributions, in dollars. AAD indicates American Academy of Dermatology; AAENT, American Academy of Otolaryngology; AAFP, American Academy of Family Physicians; AAN, American Academy of Neurology; AANS, American Association of Neurologic Surgeons; AAO, American Academy of Ophthalmology; AAOMFS, American Association of Oral and Maxillofacial Surgery; AAOS, American Association of Orthopaedic Surgeons; ACC, American College of Cardiology; ACEP, American College of Emergency Physicians; ACOG, American Congress of Obstetricians & Gynecologists; ACP, American College of Physicians Services; ACR, American College of Radiology; ACRh, American College of Rheumatology; ACS, American College of Surgeons; AMA, American Medical Association; APA, American Psychiatric Association; ASA, American Society of Anesthesiologists; ASIPP, American Society of Interventional Pain Physicians; ASPS, American Society of Plastic Surgeons; ASTRO, American Society for Radiation Oncology; CAP, College of American Pathologists; NASS, National Association of Spine Specialists; STS, Society of Thoracic Surgeons; and SVS, Society for Vascular Surgery.

## Discussion

During the 2016 election cycle, the 25 largest PACs affiliated with physician professional organizations contributed to candidates whose voting history and stances on firearm regulation did not align with evidence-based firearm policy, such as expanding background checks. These PACs contributed to more than twice as many incumbent US Senate candidates who voted against an amendment to expand firearm background checks compared with candidates who voted for the amendment. There was a similar pattern of giving by these PACs in the US House of Representatives, with candidates not cosponsoring HR 1217, a bill to expand background checks, receiving more than $2.8 million more than cosponsors. In addition, these physician PACs were more than twice as likely to contribute to and gave almost $1.5 million dollars more to candidates rated A by the NRA-PVF, a group that publicly opposes most of the evidence-based firearm policies endorsed by health care professional organizations.^15,16,17,18^

Firearm injury is a major public health problem in the United States, causing death, injury, productivity losses, and significant medical expenditures.^20^ Firearm injuries are uniquely morbid and lethal; the mean case fatality rate has been estimated at 85% for firearm-related self-harm, 19% for intentional assaults, and 5% for unintentional injuries.^20^ An estimated 10% of resulting deaths occur at the scene of the shooting, and 97% of deaths occur in the first 24 hours after injury.^21^ Because of the severity of injuries associated with firearms, many are not amenable to medical care; prevention is essential to improve health outcomes.

A number of legislative actions and public policies have been reported to effectively prevent firearm injuries.^10^ Expansion of background checks, which is supported by the general public and licensed firearm retailers,^22,23^ is associated with a reduction in suicide, homicide, and unintentional firearm injury.^24,25^ Evidence supports the association of permit-to-purchase laws, child-access prevention laws, and minimum purchasing ages with reduction in rates of firearm injuries.^10,26,27^ There is also limited evidence to support that a minimum purchasing age of 21 years may be associated with reduced suicides involving firearms among youths and to support prohibiting the sale of firearms to individuals who have previously been involuntarily committed to a psychiatric facility.^10^ Other policies, including those that seek to curb firearm trafficking, restrict firearms in public places, and ban military-style assault weapons, lack strong evidence of efficacy.^10^ However, most of these interventions have not been rigorously studied because of limitations on federally funded research, a restriction supported by the NRA-PVF.^18^

Organized physician leadership can play an important role in public health efforts to prevent firearm violence. Physicians witness first hand the physical and emotional damage experienced by patients injured by guns and have authority in advocating for health-related policy change. Many physician professional societies have embraced this role by endorsing the 2015 Call to Action, publishing policy positions on firearms, hosting advocacy training, and supporting research. However, our analysis indicates that most of the largest physician organizations’ PACs contribute more to candidates whose stances on firearm policy are in direct opposition to evidence-based firearm policies and to their organization’s stances.

Providing direct care for patients who experience firearm violence does not appear to be associated with organizations’ contribution patterns. Those PACs affiliated with the professional societies of specialties that provide frontline care for patients who experience firearm violence, including emergency medicine, general surgery (which includes trauma surgeons), orthopedic surgery, neurosurgery, anesthesiology, and radiology, gave more total dollars to incumbent candidates with records against expanding background checks in the House and the Senate, and all supported a higher proportion of candidates rated A by the NRA-PVF than those not rated A. Among the 9 organizations that endorsed the 2015 Call to Action, all but 1 gave more to candidates opposed to policies endorsed in the Call to Action. After adjusting for other political factors that influence PAC contributions, we found that endorsement of the Call to Action was associated with a smaller relative likelihood of contributing to candidates who received an A rating from the NRA-PVF than those rated B to F. Therefore, although endorsement of firearm safety policies may reflect a small difference in political giving, it does not mean that a physicians’ organization has elevated firearm policy to the level of a contribution criteria for the PAC.

The conflict between public health advocacy and political giving is not new. In 1994, Sharfstein and Sharfstein^6^ published research highlighting the discrepancy between the AMA’s call to regulate the tobacco industry and their affiliated PAC contributing nearly 3 times more money to senators who voted against a proposal that would have increased cigarette taxes and granted the US Food and Drug Administration authority to regulate tobacco than to supporters of the proposal. In a response, AMA leaders argued that “contrast[ing] public health goals and AMPAC [American Medical Political Action Committee] contributions is ‘to compare not apples and oranges but apples and hippopotamuses.’”^28^^(1614-1615)^ However, we believe that physician leadership—in the form of public statements and financial support—is important in shaping policy on public health issues. Such advocacy is more likely to be effective when political contributions are aligned with organizations’ policy statements.

### Limitations

This analysis has several limitations. First, we included only the 25 physician organization PACs with the largest total contributions not the largest organizational membership. Although there are more physician organization–affiliated PACs, we believe this is a reasonable group to analyze because the largest 25 PACs include many groups that endorsed the Call to Action and many specialties that provide initial care for patients who experience firearm violence. Of note, this group does not include some large professional organizations, such as the American Academy of Pediatricians, which does not have an affiliated PAC.

Second, use of the NRA-PVF endorsement as a measure of opposition to firearm safety legislation may be questioned. The NRA is a large organization that pursues a wide variety of activities, including gun safety education programs, such as the NRA Carry Guard, which provides comprehensive gun training to owners. The NRA-PVF, the NRA’s affiliated PAC, is a legally separate entity, with a political agenda in opposition to many evidence-based policies endorsed by the Call to Action.^15,16,17,18^ Because there have been few votes on firearm-related legislation in the US Congress recently, the NRA-PVF rating is the most comprehensive summary of candidates’ positions available.

Third, correlation is not causation. This study only examines the association between PAC contributions to political candidates and their support for firearms. It is unlikely that physician organization–affiliated PACs contribute to candidates because they are opposed to firearm regulation. Instead, physician organization–affiliated PACs consider many factors when deciding which candidates to support, such as stance on malpractice reform, physician payment policies, and the Patient Protection and Affordable Care Act as well as chance of winning and incumbent status. Some of these factors, such as stance on malpractice reform, may correlate with a candidate’s position on firearm safety regulation because support for medical malpractice reform and NRA-PVF A ratings are correlated with the Republican party.^29^ In addition, in the 2016 election cycle, Republicans had majorities in the US House of Representatives and the US Senate, and incumbency is a strong determinant of PAC contribution. Nonetheless, even if candidates’ stance on firearm safety legislation is not the reason for contribution patterns, these contributions may have the unintended consequence of helping elect candidates whose stances run counter to the organizations’ public health goals.

Fourth, few bills that propose firearm regulation have come to a vote in recent congressional sessions, which limits the power of this analysis. We analyzed the Senate and House bills that most closely aligned with the language in the Call to Action surrounding the expansion of background checks and received the most legislative activity and found differential contribution patterns.

Fifth, classification of lack of cosponsorship for HR 1217 as opposition to firearm safety legislation can be questioned because candidates could have supported the legislation without cosponsoring. We think cosponsorship is a reasonable proxy for support because this amendment was the most high-profile firearm safety bill in the House during the 114th US Congress.

## Conclusions

Gun violence is the leading cause of preventable injury and death in the United States. National physician professional organizations are well positioned to lead in advocating for public policies that reduce firearm-related morbidity and mortality, and many have taken public positions in favor of such policies. However, our findings suggest that these organizations’ associated PACs contribute more often and give more money to candidates who oppose evidence-based firearm regulation than to those who support it. This donation pattern appears to be inconsistent with and a barrier to effective public health advocacy for firearm safety.

## Notes

Supplement. **eAppendix 1.** Organizational Policies on Firearm Regulation of Physician Organizations Affiliated With 25 Largest PACs, as of Election Day 2016 **eAppendix 2.** Description of National Rifle Association Political Victory Fund (NRA-PVF) Ratings **eAppendix 3.** Odds Ratios of Support of A-Rated Candidates by All Top 25 Physician Organization-Affiliated PACs, Adjusting for Endorsement Status and Other Confounders
