# Supplementary material for: Association of Physician Organization–Affiliated Political Action Committee Contributions With US House of Representatives and Senate Candidates’ Stances on Firearm Regulation
Source: JAMA Netw Open. 2019 Feb 22;2(2):e187831. doi: 10.1001/jamanetworkopen.2018.7831 (PMC6484593; doi:10.1001/jamanetworkopen.2018.7831)
Supplement: Supplement. — eAppendix 1. Organizational Policies on Firearm Regulation of Physician Organizations Affiliated With 25 Largest PACs, as of Election Day 2016 eAppendix 2. Description of National Rifle Association Political Victory Fund (NRA-PVF) Ratings eAppendix 3. Odds Ratios of Support of A-Rated Candidates by All Top 25 Physician Organization-Affiliated PACs, Adjusting for Endorsement Status and Other Confounders [file jamanetwopen-2-e187831-s001.pdf]

Supplementary Online Content

Schuur JD, Decker H, Baker O. Association of physician organization–affiliated political action committee contributions with US House of Representatives and Senate candidates' stances on firearm regulation. *JAMA Netw Open*. 2019;2(2):e187831. doi:10.1001/jamanetworkopen.2018.7831

eAppendix 1. Organizational Policies on Firearm Regulation of Physician Organizations Affiliated With 25 Largest PACs, as of Election Day 2016

eAppendix 2. Description of National Rifle Association Political Victory Fund (NRA-PVF) Ratings

eAppendix 3. Odds Ratios of Support of A-Rated Candidates by All Top 25 Physician Organization-Affiliated PACs, Adjusting for Endorsement Status and Other Confounders

This supplementary material has been provided by the authors to give readers additional information about their work.

© 2019 Schuur JD et al. *JAMA Network Open*.

eAppendix 1. Organizational Policies on Firearm Regulation of Physician Organizations Affiliated With 25 Largest PACs, as of Election Day 2016

Physician Professional Organization	Affiliated PAC Official FEC Name*	Wrote or Endorsed 2015 Call to Action?**	Specific Policy on Firearm Regulation?	Recommendation Content
American Association of Orthopaedic Surgeons	Political Action Committee of the American Association of Orthopaedic Surgeons	No	No	*Firearms Violence (Retired September 2010)*
American Medical Association	American Medical Association Political Action Committee	Endorsed	Firearms as a Public Health Problem in the United States - Injuries and Death H-145.997	Support for waiting periods and background checks for all firearm purchasers<br><br>Stricter enforcement of present federal and state gun safety legislation, and the imposition of mandated penalties for crimes committed with the use of a firearm, including the illegal possession of a firearm<br><br>Increased research on firearm safety
American Society of Anesthesiologists	American Society of Anesthesiologists Political Action Committee	No	No	--
American College of Emergency Physicians	National Emergency Medicine Political Action Committee	Original Author	Firearm Safety and Injury Prevention	Encourage the change of societal norms that glorify a culture of violence<br><br>Increased public and private funding for firearm safety and injury prevention research, including on the effect of socioeconomic and other cultural risk factors<br><br>Create a confidential national firearm injury research registry while encouraging states to establish a uniform approach to tracking and recording firearm related injuries<br><br>Promote access to effective, affordable, and sustainable mental health services<br><br>Protect the duty of physicians and encourage health care provider discussions with patients on firearm safety<br><br>Promote the development of technology that increases firearm safety<br><br>Support universal background checks for firearm transactions<br><br>Require the enforcement of existing laws and support new legislation that prevents high risk and prohibited individuals from obtaining firearms by any means

© 2019 Schuur JD et al. *JAMA Network Open*.

				Restrict the sale and ownership of weapons, munitions, and large-capacity magazines that are designed for military or law enforcement use
American College of Radiology	American College of Radiology Association PAC	No	No	--
American Academy of Dermatology	American Academy of Dermatology Association Political Action Committee (SKINPAC)	No	No	--
American Academy of Ophthalmology	American Academy of Ophthalmology Inc Political Committee (OPTHPAC)	No	No	--
American College of Surgeons	American College of Surgeons Professional Association PAC	Original Author	Statement on Firearm Injuries	Promote legislation banning civilian access to assault weapons, large ammunition clips, and munitions designed for military and law enforcement agencies<br><br>Enhancing mandatory background checks for the purchase of firearms to include gun shows and auctions<br><br>Ensuring that health care professionals can fulfill their role in preventing firearm injuries by health screening, patient counseling, and referral to mental health services for those with behavioral medical conditions<br><br>Developing and promoting proactive programs directed at improving safe gun storage and the teaching of non-violent conflict resolution for a culture that often glorifies guns and violence in media and gaming<br><br>Evidence-based research on firearm injury and the creation of a national firearm injury database to inform federal health policy
American Academy of Family Physicians	American Academy of Family Physicians Political Action Committee	Original Author	Firearms and Safety Issues	Increased research into all areas of how gun violence affects public health, including but not limited to, research into the epidemiology, prevention, safety and risks related to gun violence in the United States<br><br>Strong and robust enforcement of existing federal, state, and local laws and regulations regarding the manufacture, sale and possession of guns, including increased efforts to enforce current laws on illegal gun trafficking<br><br>Legislation restricting unsupervised access to both firearms and ammunition by children.<br><br>Opposition of private ownership of weapons designed primarily to fire multiple (greater than 10) rounds quickly

© 2019 Schuur JD et al. *JAMA Network Open*.

American Congress of Obstetricians & Gynecologists	The American Congress of OB-GYNS PAC	Original Author	Gun Violence and Safety	Routine screening for intimate partner violence<br><br>Opposes governmental restrictions or requirements dictating the content of physician/patient counseling regarding firearms<br><br>Encourages the appropriate federal and state agencies to support and fund research, surveillance activities, public education, and anti-violence initiatives that recognize and address the role of intimate partner violence and firearms in the health and safety of women<br><br>Supports laws or regulations limiting the purchase and ownership of firearms by individuals with emergency, temporary, or permanent protective or restraining orders or those with intimate partner violence and/or stalking convictions<br><br>Encourages improved access to mental care and services
American Academy of Neurology	American Academy of Neurology (BRAINPAC)	Endorsed	No	--
American College of Cardiology	American College of Cardiology Political Action Committee	No	No	--
American Association of Oral and Maxillofacial Surgery	American Association of Oral And Maxillofacial Surgeons Political Action Committee	No	No	--
American Psychiatric Association	American Psychiatric Association Political Action Committee	Original Author	Position Statement on Firearm Access, Acts of Violence and the Relationship to Mental Illness and Mental Health Services	Requiring background checks and waiting periods on all gun sales or transactions<br><br>Requiring safe storage of all firearms in the home, office, or other places of daily assembly<br><br>Regulating the characteristics of firearms to promote safe use for lawful purposes and to reduce the likelihood that they can be fired by anyone other than the owner without the owner's consent<br><br>Banning possession of firearms on the grounds of colleges, hospitals, and similar institutions by anyone other than law enforcement and security personnel<br><br>Assuring that physician and other health care professionals are free to make clinically appropriate inquiries of patients and others about possession of and access to firearms and take necessary steps to reduce the risk of loss of life by suicide, homicide, and accidental injury<br><br>Prioritize research on the causes of firearm violence and its effective control<br><br>Reasonable restrictions on gun access are appropriate, but not restrictions based solely on a diagnosis of mental disorder

© 2019 Schuur JD et al. *JAMA Network Open*.

				Improved identification and access to care for persons with mental disorders may reduce the risk of suicide<br>and violence involving firearms for persons with tendencies toward those behaviors
College of American Pathologists	College of American Pathologists Political Action Committee	No	No	--
American Society of Plastic Surgeons	American Society of Plastic Surgeons (PLASTYPAC)	No	No	--
American College of Rheumatology	American College of Rheumatology (RHEUMPAC)	No	No	--
American Academy of Otolaryngology	American Academy Of Otolaryngology-Head And Neck Surgery (ENT PAC)	No	No	--
Society of Thoracic Surgeons	Society of Thoracic Surgeons Political Action Committee	Endorsed	No	--
American Society for Radiation Oncology	American Society for Radiation Oncology PAC (ASTRO-PAC")	No	No	--
American College of Physicians Services	American College of Physician Services Inc PAC	Original Author	Reducing Firearm-Related Injuries and Deaths in the United States: Executive Summary of a Policy Position Paper From the American College of Physicians	Adopt a public health approach to firearms-related violence and the prevention of firearm injuries and deaths<br><br>Support for universal background checks, waiting periods for firearm purchases, a ban on firearms that are undetectable by metal detectors or standard security screening devices<br><br>Recommends that guns be subject to consumer product regulations regarding access, safety, and design<br><br>Ensuring adequate access to mental health services<br><br>Support for legislation to ban the sale and manufacture for civilian use of firearms that have features designed to increase their rapid killing capacity (often called “assault weapons” or semiautomatic weapons) and large-capacity ammunition.<br>Support for efforts to improve and modify firearms to make them as safe as possible, including the incorporation of built-in safety devices (such as trigger locks and signals that indicate a gun is loaded)<br><br>Support for increased research on firearm violence and on intervention and prevention strategies to reduce injuries caused by firearms. The Centers for Disease Control and Prevention, National Institutes of Health, and National Institute of

© 2019 Schuur JD et al. *JAMA Network Open*.

				Justice should receive adequate funding to study the impact of gun violence on the public's health and safety. Access to data should not be restricted
American Association of Neurologic Surgeons	American Association of Neurological Surgeons Political Action Committee (NEUROSURGERYPAC)	No	No	--
American Society of Interventional Pain Physicians	American Society of Interventional Pain Physician PAC	No	No	--
National Association of Spine Specialists	Society for Vascular Surgery Political Action Committee	No	No	--
Society for Vascular Surgery	Spine PAC of the National Association of Spine Specialists	No	No	--

Abbreviations: PAC = Political Action Committee; FEC = Federal Election Commission.

*Source: Opensecrets.org.

**Source: Firearm-Related Injury and Death in the United States: A Call to Action From Over 50 Supportive Organizations and the American Bar Association.

© 2019 Schuur JD et al. *JAMA Network Open*.

eAppendix 2. Description of National Rifle Association Political Victory Fund (NRA-PVF) Ratings

**NRA-PVF Grade**	**Meaning**
A+	A legislator with not only an excellent voting record on all critical NRA issues, but who has also made a vigorous effort to promote and defend the Second Amendment.
A	Solidly pro-gun candidate. A candidate who has supported NRA positions on key votes in elective office or a candidate with a demonstrated record of support on Second Amendment issues.
A_Q	A pro-gun candidate whose rating is based solely on the candidate's responses to the NRA-PVF Candidate Questionnaire and who does not have a voting record on Second Amendment issues.
B	A generally pro-gun candidate. However, a "B" candidate may have opposed some pro-gun reform or supported some restrictive legislation in the past.
C	Not necessarily a passing grade. A candidate with a mixed record or positions on gun related issues, who may oppose some pro-gun positions or support some restrictive legislation.
D	An anti-gun candidate who usually supports restrictive gun control legislation and opposes pro-gun reforms. Regardless of public statements, can usually be counted on to vote wrong on key issues.
F	True enemy of gun owners' rights. A consistent anti-gun candidate who always opposes gun owners' rights and/or actively leads anti-gun legislative efforts, or sponsors anti-gun legislation.
?	Refused to answer the NRA-PVF Candidate Questionnaire, often an indication of indifference, if not outright hostility, to gun owners' and sportsmen's rights.

***Source**: Grades and Endorsements: National Rifle Association website.

© 2019 Schuur JD et al. *JAMA Network Open*.

eAppendix 3. Odds Ratios of Support of A-Rated Candidates by All Top 25 Physician Organization-Affiliated PACs, Adjusting for Endorsement Status and Other Confounders.

**Variable**	**ALL 25 PACs (Odds Ratios and 95% CI)**
**PAC Contribution Amount >0**	
A-Rated Indicator	2.05 (0.91, 4.62)
Endorsed Call to Action Indicator (Y/N)	2.18 (1.19, 3.97)
A-Rated * Endorsed	0.76 (0.57, 0.99)
Incumbent Dummy	5.58 (2.52, 12.37)
Vote Share 40-55% Dummy	19.96 (5.77, 68.99)
Vote Share 55-75% Dummy	34.27 (8.91. 130.76)
Vote Share >75%	29.59 (7.47, 117.14)
Republican	0.61 (0.27, 1.37)

* Standard errors have been clustered by PAC and Candidate ID

Note: The dataset on which the regression coefficients are computed is at the candidate-PAC level. Each candidate has 25 rows, one for each PAC. Each PAC has 751 rows, one for each candidate. Therefore, standard errors must be clustered both at the PAC and at the candidate level as the observation within each candidate and each PAC are correlated. Standard code for logistic regression in Stata does not allow clustering on more than one variable. We used the logit2 command written by Mitchell A. Petersen at the Kellogg School of Management at Northwestern University. This code allows for two-way clustering for non-nested variables.

Source: http://www.kellogg.northwestern.edu/faculty/petersen/htm/papers/se/se_programming.htm

Stata Code: logit2 PAC_Yes NRARatingA endorsed_policy NRARatingA_endorsed incumbent VoteShare4055 VoteShare5575 VoteShare75plus Republican, tcluster(PAC) fcluster(CandidateID)

© 2019 Schuur JD et al. *JAMA Network Open*.
